# Outcome of the Pediatric Patients with Portal Cavernoma: The Retrospective Study for 10 Years Focusing on Recurrent Variceal Bleeding

**DOI:** 10.1155/2016/7953870

**Published:** 2016-02-02

**Authors:** Hongjie Guo, Fabao Hao, Chunbao Guo, Yang Yu

**Affiliations:** ^1^Department of Pediatric General Surgery and Liver Transplantation, Children's Hospital, Chongqing Medical University, Chongqing 400014, China; ^2^Department of Pediatric Surgery, Children's Hospital of Jinan, Shandong University, Jinan 250000, China; ^3^Ministry of Education Key Laboratory of Child Development and Disorders, Children's Hospital, Chongqing Medical University, Chongqing 400014, China

## Abstract

*Background*. Portal cavernoma (PC) is the most critical condition with risk or variceal hemorrhage in pediatric patients. We retrospectively investigated the patients with PC focusing on the predictors for recurrent variceal bleeding.* Methods*. Between July 2003 and June 2013, we retrospectively enrolled all consecutive patients admitted to our department with a diagnosis of PC without abdominal malignancy or liver cirrhosis. The primary endpoint of this observational study was recurrent variceal bleeding. Independent predictors of recurrent variceal bleeding were identified using the logistic regression model.* Results*. A total of 157 patients were enrolled in the study. During the follow-up period, 24 patients exhibited onset of recurrent variceal bleeding. Acute variceal bleeding was subjected to conservative symptomatic treatment and emergency endoscopic sclerotherapy. Surgical procedure selection was based on the severity of vascular dilation and collateral circulation. Multivariate logistic regression analysis demonstrated that the presence of ascites, collateral circulation, and portal venous pressure were independent prognostic factors of recurrent variceal bleeding for patients with portal cavernoma.* Conclusions*. The presence of ascites, collateral circulation, and portal venous pressure evaluation are important and could predict the postsurgical recurrent variceal bleeding in patients with portal cavernoma.

## 1. Introduction

Portal cavernoma (PC), also known as cavernous transformation of the portal vein, plays an important role in gastrointestinal bleeding among children. At least one episode of upper gastrointestinal bleeding was present in approximately 80% of patients with PC [1]. The bleeding episodes are associated with frequent hospital admissions and high morbidity. PC usually is characterized with an extended network of small and extremely sinuous vessels in the hilum to compensate for the impaired hepatofugal flow, resulting from a portal vein (PV) thrombotic event. Slow blood flow through this tortuous network of veins, redistribution of the blood, and new thromboses all contribute to the increase of portal vein pressure [2–4]. Following this pathophysiological condition, portal hypertension might eventually develop with esophagogastric varices, splenomegaly, and even biliary abnormalities in the majority of patients with PC. The optimal therapeutic strategy for esophagogastric varices and variceal bleeding is multifaceted and controversial and so represent a difficult therapeutic problem. Given the complexity and rarity of the portal cavernoma, a variety of surgical techniques, such as various portosystemic shunts and gastrosplenic decompression, have been proposed to focus on the prevention and treatment of variceal hemorrhage, prevention of recurrent thrombosis, and treatment of symptomatic portal biliopathy [5, 6]. Up to now, the totally agreed upon guidelines for this disease are unavailable, and therapeutic options vary in different centers; when these fail, further medical options are limited, especially for intrahepatic cavernoma.

Because treatment is currently inadequate, it is imperative that a better understanding of the predictors for recurrent variceal bleeding of PC is gained to identify new targets for therapeutic intervention. Previous studies have reported that 20-year survival rate in patients is closely associated with advanced age, presence of malignancy and cirrhosis, high bilirubin, and deterioration of liver function [7]. However, most long-term survivors develop complications; at the same time, little information is known regarding the prognostic factors in pediatric patients with portal cavernoma due to its low morbidity and mortality. Until the age of 18, biliary atresia (BA) patients are generally managed by pediatricians or pediatric surgeons. After the age of 18 these patients are referred to and managed by gastroenterologists. It is therefore important that gastroenterologists become aware of the fact that, in long-term survivors who have undergone the variceal hemorrhage operation, complications such as recurrent variceal hemorrhage and portal hypertension may occur.

The aim of this research is to retrospectively study the predictors for recurrent variceal bleeding in pediatric patients who undergone operation during childhood after a follow-up of 5 years by a uniform therapeutic strategy at our center. This research attempts to evaluate the most important predictors for recurrent variceal hemorrhage to optimize the therapeutic selection.

## 2. Materials and Methods

### 2.1. Subject Characteristics

This study was performed in accordance with the ethical standards prescribed by the Helsinki Declaration of the World Medical Association and approved by the Institutional Review Board of the Chongqing Medical University and Shandong University. The Children's Hospital of Jinan and Chongqing Medical University have the capacity of 1500 beds and could provide tertiary care in southwest China and Shandong province. Department of hepatobiliary surgery is a level III, 50-bed ward and has 1000–5000 admissions per year. From January 2003 to July 2013, 167 consecutive patients with confirmed diagnosis of portal cavernoma were enrolled in this observational study. All subjects were ethnic Chinese. Patients were excluded if they had abdominal malignancy or liver cirrhosis. Additionally, these patients would be excluded if they were receiving experimental treatment trial or were unable to have regular hepatic function assessments. The clinical records were thoroughly collected upon admission or referral. Regular blood tests, hepatic tests, prothrombin time, internationalized normalized ratio (INR), color doppler ultrasound (CDUS), and endoscopy were performed in all patients. After 2007, 64-slice computed tomography (CT) was available in our hospital, so it was performed in the latest 83 cases.

### 2.2. Condition Evaluation

Under the 64-slice CT measurement, portal cavernoma was visualized with a tortuous network of veins in the hilum. The degree of ascites was divided into heavy, medium, and modicum under the CDUS evaluation. Hypersplenism was defined as a low WBC count of 1520 cells/mm^3^ and a low platelet count of 32000/mm^3^. Splenomegaly was diagnosed by palpation 6 cm below the left costal margin. The portal hypertension at admission was defined as the presence of splenomegaly and/or esophageal varices, even exhibited with ascites.

### 2.3. Therapeutic Strategy

The therapeutic strategy was to gain the maximal beneficial effects of symptom resolution with minimal invasiveness. Conservative symptomatic treatment was used in patients with no obvious symptoms or little bleeding. Acute variceal bleeding was treated with urgent medical or endoscopic therapy. Prophylactic endoscopic therapy was selected for high-risk varices. The surgical procedure was considered if patients presented with a long history of repeated gastrointestinal syndromes unresponsive to conservative therapies. The surgical procedures were modified according to the location of the cavernous transformation and the clinical manifestation.

### 2.4. Follow-Up Management

The patients were followed up every 3 months during the first 3 years after diagnosis and then every 6 months. The follow-up content included the incidence of hematemesis and melena after surgery, as well as clinical examination, basic laboratory tests, and color doppler ultrasound examination. Information on clinical, laboratory, and imaging examinations, as well as treatment procedures and prognosis, was recorded for all patients. The treatments and their effectiveness were evaluated for each patient. The median follow-up time in our study was 55 months (from 3 mo to 11 years).

### 2.5. Statistical Analysis

Statistical comparisons were carried out by SPSS software for Windows (SPSS, Chicago, IL). Quantitative data were reported as mean ± SEM and were compared with the independent sample *t*-test or one-way analysis of variance; Pearson's *χ* ^2^ test or Fisher's exact test was used to examine correlations between the procedures and clinicopathological features reported as frequencies. Univariate and multivariate logistic regression models were used to examine the independent predictors of recurrent variceal bleeding. The covariates incorporated into the multivariate analysis were the variables that reached *P* < 0.1 in univariate analysis. Two-tailed *P* values < 0.05 were considered statistically significant.

## 3. Results

### 3.1. Patient Characteristics

At the time of analysis, 167 eligible and evaluable patients diagnosed with portal cavernoma without liver cirrhosis or abdominal malignancy fulfilled the criteria for inclusion in our study. Ten patients were excluded from the study because they lacked proper documentation.  Table 1 shows the patients' clinical characteristics at inclusion in the study. Among them, 101 of the 157 (64.3%) patients presented their first variceal bleeding episode and 13 of 157 (8.3%) patients presented their second variceal bleeding episode without treatment. Severe ascites were found by color doppler ultrasound in three patients.

### 3.2. Preoperative Angiography Evaluation

**Figure 1:**
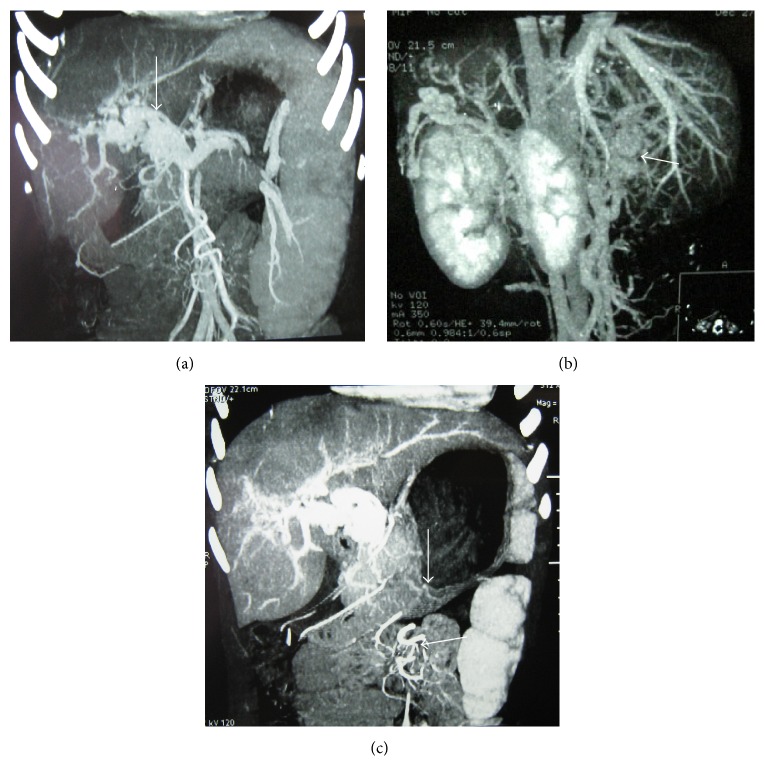
Dilated and tortuous portal vein cavernoma. Axial and coronal of sixty-four-slice CT angiography indicate (a) dilated and tortuous portal vein, (b) intrahepatic PV, and (c) collateral circulation (arrow).

The lesion location and portal vein-vena cava shunt on angiography are summarised in Table 2. The normal PV structure disappeared at the hepatic hilar area for the 57 pediatric patients with PC under angiography measurement and different degrees of dilation indicated in the major branches of the PV (Figure 1). Several cases exhibited severe dilated blood vessels in the lower esophagus and under the gastric fundic mucosa with lumpy, tortuous of the lumen of the lower esophagus and lumpy protuberance in the gastric cavity (Figure 1(a)). Liver laceration in the intrahepatic PV (narrowing or occlusion, or unclear angiograms of the left and/or right branches) was detected in three cases (Figure 1(b)). Eight cases exhibited spontaneous splenorenal/gastrorenal shunt (highly tortuous, dilated blood vessel structure). Nine cases presented with open retroperitoneal communicating branches (venous plexus of Retzius), showing tortuous disordered retroperitoneal blood vessels (in a bundle shape or cirsoid shape) connected to the inferior vena cava. Seven cases presented paraumbilical vein patefaction, showing tortuous dilation changes in the ligamentum teres hepatis. The blood vessels were in a radial pattern and connected to chest wall veins or the deep and superficial veins of the abdominal wall (Figure 1(c)).

### 3.3. Surgical Features

Of the patients with acute variceal bleeding, 35 received pharmacological treatment, 18 had emergency endoscopic sclerotherapy, and 13 underwent ligation of the gastric varices via a prophylactic endoscopic approach (Table 3). Active bleeding was controlled in these patients. Surgical procedure selection was based on overall consideration of several factors, according to the severity of vascular dilation, the PC location, and the extent of liver dysfunction. Splenectomy was performed for 21 cases with apparent splenomegaly, but without obvious lumpy, tortuous dilation of the lower esophagus and gastric fundus veins. Surgical vascular disconnection in the gastric fundus and lower esophagus in combination with splenectomy was performed in 36 cases with severe tortuous dilation in the lower esophagus and gastric fundic mucosa. Among them, surgical thrombus removal and end-to-end anastomosis of the PV were performed in 8 cases with the main PV trunk occlusion. In three children tortuous dilation of the intrahepatic portal vein, with severe damaged liver function, was detected. Living-donor liver transplantation was selected for these patients.

Splenorenal shunt was attempted for the patients with recurrent variceal bleeding in eight patients. The 2 patients with splenorenal shunt failure experienced at least two episodes of rebleeding within 6 mo, and 1 was lost to follow-up. The collateral circulation formation should be considered for selecting surgical procedures, which can help estimate prognosis on postoperative recurrence of gastrointestinal bleeding.

### 3.4. Comparison of Patients with and without Postprocedural Recurrent Variceal Bleeding

A comparison of patients with and without* postprocedural *recurrent variceal bleeding is given in Table 4. Of the patients with procedure, 24 (15.3%) developed recurrent variceal bleeding. There were 32 episodes of recurrent variceal bleeding in these 24 patients. Five patients had two episodes of recurrent variceal bleeding. The median duration from the time of intubations to the onset of recurrent variceal bleeding was 11.2 ± 5.7 months. Among the 24 patients with recurrent variceal bleeding, 3 patients were transferred to other hospitals.

In the univariate analysis, variables that were significantly associated with recurrent variceal bleeding were indicated in Table 4. Compared to patients without recurrent variceal bleeding, those with recurrent variceal bleeding had a longer history of portal cavernoma (1.11 ± 0.87 years in the nonrecurrent bleeding group versus 4.53 ± 2.81 years in the bleeding group, *P* = 0.003). A fibrotic cord replacing the main portal vein, the collateral circulation in the lower esophagus, and gastric fundal varices were more frequently found on CT scans in patients with recurrent variceal bleeding than those without (24/24 versus 21/33, *P* < 0.01). Although collateral circulation formed at other locations can help to reduce PV pressure and was thus preserved during surgery, it indeed opened followed with the high portal venous pressure. Consistently, collateral circulation formed at other locations was more frequently observed in patients with variceal bleeding than those without (11/24 versus 1/33, *P* < 0.01). As expected, significant relationship between portal venous pressure and variceal bleeding was also observed (Table 2). Variables that were significantly associated with recurrent variceal bleeding in univariate analysis and had a *P* value less than 0.1 were entered into a multivariable regression analysis. The presence of severe ascites, collateral circulation, and high portal venous pressure remained significant in the logistic regression model and so were regarded as independent predictors of recurrent variceal bleeding in pediatric patients with portal cavernoma (Table 5).

### 3.5. Follow-Up

The average time of follow-up in this cohort was 6 years (from 3 mo to 11 years). During the monthly follow-up visits at the outpatient clinic, 9 patients exhibited recurrent variceal bleeding and experienced the first episode of rebleeding within 6 mo, one patient presented with recurrent variceal bleeding within one year after operation and 14 patients experienced at least one episode of rebleeding a year later. One patient died of massive variceal rebleeding 49 days after discharge. Following the splenectomy and gastric fundic and lower esophageal vascular disconnection, no severe hematemesis recurrence or obvious black tarry feces presented in 33 pediatric patients. In 5 patient, partial recanalization of the main portal vein was found and no obvious deterioration of tortuous dilation changes in main portal vein were detected in 31 cases, while, in the remaining 21 patients, the main portal vein became cavernous collateral vessels and unidentifiable. One patient experienced melena 2 wk after being discharged from hospital. Under barium meal examination six months after surgery, esophageal varices disappeared in 10 cases and were relieved in one and one patient was lost to follow-up. One patient developed adhesive ileus two wk after surgery and was cured after another operation. Three of the patients received living-donor liver transplantation; among them, one died of portal thrombosis 5 d after surgery. The other two patients recovered well during the follow-up.

## 4. Discussion

In this study, we performed a retrospective cohort study to evaluate the outcome of pediatric patients with portal cavernoma and measure predictors of recurrent variceal bleeding after medical intervention. Our study demonstrated that most patients with portal cavernoma had a relatively benign course. In this study, we also found several potentially amenable risk factors, which can offer prognostic information about the probability of developing* postprocedural* recurrent variceal bleeding in individual patients and may lead to development of effective preventive strategies. We confirmed that severe varices, collateral circulation, and portal venous pressure were independently risk factors for the development of recurrent variceal bleeding by stepwise logistic regression. To our knowledge, this is the largest study to describe prognostic information and the outcome of portal cavernoma patients in pediatrics.

Preoperative understanding of the degree and size of the CTPV is very important for the surgical process [8, 9] or prognosis [10]. The condition of CTPV has an apparent impact on the difficulty level of operation, incidence of postoperative complications, and long-term health status [11, 12]. We found that coronary venous reflux was a significant risk factor for recurrent variceal bleeding. Coronary venous reflux is an indicator of esophageal variceal rupture, the important pathological symptom of portal hypertension. In the present study, collateral vessels in the patients in the present study were mainly located in the lower esophagus and gastric fundus, which was consistent with the clinical symptoms of upper gastrointestinal bleeding such as hematemesis and melena. The patients with the most severe hematemesis presented with tortuous and stiffness in left gastric veins in 64-slice CT scan. In addition, collateral vessels from the open umbilical vein and splenorenal vein to the superior and inferior vena cava were found to reduce the pressure of the gastric coronary vein. Also, identification of RVs has been demonstrated more frequently in our patients with recurrent variceal bleeding, indicating the high portal pressure [13–15]. In the present study, open RV was found in nine cases, and spontaneous paraumbilical vein patefaction was found in three. Although all these collateral vessels were conserved during surgery, most of the patients displayed upper gastrointestinal bleeding after surgery. In the present study, three cases presented hemangioma-like blood vessels in the intrahepatic portal system resulting in damaged liver function and hypersplenism through biliary compression, which is an indication for liver transplantation. Furthermore, we found that the presence of ascites was another important prognostic factor for the posttreatment recurrent variceal bleeding. This finding could be explained by the higher incidence of portal pressure in patients with ascites [16, 17]. Indeed, the presence of ascites is caused not only by the portal pressure itself, but also by a deterioration of liver function and a lower level of serum albumin and sodium, which are closely correlated with the presence of ascites [18, 19]. Based on the importance of ascites in pediatric patients with portal cavernoma, therapeutic decision making needs to be altered according to the presence of ascites [20]. We should pay more attention to the underlying liver dysfunction and comorbidities. Care and correction of liver dysfunction and its comorbidities, like lower serum albumin and sodium, should be incorporated into the treatment strategy for portal cavernoma.

Although the recurrent variceal bleeding was demonstrated with high prevalence in our patients, the post-medical intervention death was not so prominent (only one patient died of massive variceal bleeding), which could be explained by the advances in bleeding control and long-term liver function maintenance [21–23]. We found that the death resulting from sudden and massive variceal bleeding always occurred in the short term after medical intervention of PC, which is in accordance with report by others [24]. We inferred that the development of portal cavernoma is a tardiness and benign course. In most of the patients, the liver functions were satisfactory; the high portal vein pressure is the prominent problem to control. If we focus on this thorny issue, we could control the recurrent bleeding in a steady status. As a primary cause of death, massive variceal bleeding and its secondary severe complications often occur at the early stage [25, 26]. In our study, two patients who underwent recurrent variceal bleeding died 5 months after surgery. In few patients, the underlying disease or liver dysfunction had already become severe; we selected to perform liver transplantation in these three patients [1]. As shown previously [27], the overall mortality of splanchnic vein thrombosis patients with intestinal infarction is high in adult patients; we did not detect this complication in our cases.

As multiple factors could affect the recurrent variceal bleeding and therapeutic interventions were not used in a randomized manner in our study, we acknowledge that there are several limitations to our study. First, this is a retrospective, single-center study. Second, our database does include different therapeutic interventions that patients received. Third, the results of this study were based on an intent-to-treat analysis. Currently, there is no reference standard for PC intervention, so the inclusion criteria may influence the application of indicators in patients with variceal bleeding. This limitation was minimized because we used a definition of VAP that was established through adjudication of all suspected cases and that incorporated portal vein pressure evaluation and radiographic and clinical criteria. Due to the excellent outcome of pediatric patients with PC, the follow-up period was relatively not enough to meet the endpoints, which might depress its resolving power even miss other potential predictors. An extended follow-up research might be performed in the future. Given the descriptive design, which risk factors we found causal cannot be distinguished currently. The relative smaller sample size also restricts the detection of possible independent risk factors for recurrent bleeding and mortality. Indeed, in the multivariate analysis, the variables might introduce the risk of overfitting the data, which might bring about false positive results. Therefore, the current conclusions should be adopted cautiously and confirmed in larger research in the future.

In conclusion, our study suggests that portal angiography can effectively demonstrate the pathological changes in the PV system, especially collateral circulation, which could provide accurate information for clinical manifestation. The presence of ascites and portal venous pressure are also significantly associated with increased mortality in pediatric patients with portal cavernoma. We acknowledge that these results were taken based on a small size of patient number. Thus, large-scale multicenter clinical trials should be performed in the future to confirm the predictors in these patients and to establish the potential therapeutic strategy based on the presence of these factors.

## 5. Conclusions

We find that the presence of ascites, collateral circulation, and portal venous pressure were independent prognostic factors of postprocedural recurrent variceal bleeding for patients with portal cavernoma. So, the presence of ascites, collateral circulation, and portal venous pressure evaluation are important and could predict the postsurgical recurrent variceal bleeding in patients with portal cavernoma.

## Figures and Tables

**Table 1:** Clinical and analytical characteristics of the patients at first admission.

Age (years)	8.2 ± 3.6
Sex (M/F)	89/68
Clinical presentations	
Variceal bleeding	131
Abdominal distension	97
Splenomegaly	68
Melena	123
Degree of varices (heavy/medium/modicum/no)	11/28/66/52
Hypersplenism	79
Laboratory tests	
PT (s)	14.14 ± 0.24
WBC (10^9^/L)	8.11 ± 1.03
INR	1.15 ± 0.02
*γ*-GGT (U/L)	41.16 ± 5.96
Serum Na (mmol/L)	132.54 ± 2.32
Serum K (mmol/L)	3.9 ± 1.15

Values are expressed as the means ± standard deviation.

**Table 2:** Assessment of manifestation and portal vein-vena cava shunt on angiography.

Location of lesion	
Main portal vein	38
Intrahepatic PV	3
Collateral circulation	
Lower esophagus and gastric fundal varices	45
Spontaneous splenorenal/gastrorenal shunt	8
Paraumbilical vein patefaction	3
Venous plexus of Retzius	9

**Table 3:** Summary of therapeutic selection.

Conservative symptomatic treatment	35
Endoscopic therapy	31
Emergency endoscopic sclerotherapy	18
Prophylactic endoscopic approach	13
Albumin and/or diuretics	129
Surgical procedure	
Splenectomy	65
Splenectomy + vascular disconnection	41
Liver transplantation	3
Splenorenal shunt	8

**Table 4:** Univariate analysis of factors involved in postprocedural recurrent variceal bleeding.

Recurrent variceal bleeding	With (24)	Without (33)	*P*
Age (years)	7.8 ± 2.9	8.3 ± 3.9	0.351^b^
Sex (M/F)	15/9	24/9	0.564^b^
Clinical presentations			
Variceal bleeding 51	24	27	0.0357^b^
Abdominal distension	11	6	0.0394^b^
Splenomegaly	20	18	0.0017^b^
Melena	23	20	0.0039^b^
Degree of ascites (heavy/medium/modicum/no)	3/7/8/10	0/4/8/17	
Laboratory tests			
Hb (g/L)	89.2 ± 7.2	113.4 ± 6.8	0.0136^a^
RBC (10^12^/L)	3.28 ± 0.79	4.13 ± 0.68	0.0374^a^
PLT (10^9^/L)	226.5 ± 42.8	325.4 ± 46.7	0.0725^a^
PT (s)	15.3 ± 0.42	13.6 ± 0.52	0.1365^a^
WBC (10^9^/L)	6.7 ± 1.1	8.9 ± 1.3	0.0109^a^
INR	1.13 ± 0.02	1.16 ± 0.03	0.0914^a^
*γ*-GGT (U/L)	42.5 ± 6.9	40.6 ± 4.8	0.246^a^
Serum Na (mmol/L)	130.2 ± 3.8	135.1 ± 2.9	0.152^a^
Serum K (mmol/L)	3.5 ± 1.1	4.3 ± 1.2	0.0654^a^
Portal venous pressure (cm H_2_O)	33.8 ± 9.1	23.8 ± 6.7	0.006^a^
Manifestation on angiography			
Main portal vein	15	23	
Right portal vein obstruction	5	3	
Left portal vein obstruction	2	3	
Splenic vein obstruction	3	1	
Superior mesenteric vein obstruction	2	0	
Collateral circulation			
Lower esophagus and gastric fundal varices	24	21	0.0006^b^
Spontaneous splenorenal/gastrorenal shunt	5	1	
Paraumbilical vein patefaction	3	0	
Venous plexus of Retzius	8	1	
Total	11	2	0.0008^b^

Quantitative values are expressed as the means ± SEM. ^a^One-way ANOVA. Frequencies data were examined using Pearson's *χ* ^2^  test or Fisher's exact test^b^.

**Table 5:** Multiple logistic regression of factors associated with postprocedural recurrent variceal bleeding.

Factors	OR	95% CI	*P*
Ascites	3.46	1.25–12.72	0.009
Collateral circulation	1.63	0.68–4.52	0.031
Portal venous pressure	8.21	1.72–73.84	0.002

OR: odds ratio; CI: confidence interval.
